# Functional Training and Blood Flow Restriction: A Perspective View on the Integration of Techniques

**DOI:** 10.3389/fphys.2020.00817

**Published:** 2020-07-31

**Authors:** Marzo E. Da Silva-Grigoletto, Ezequias Pereira Neto, David George Behm, Jeremy P. Loenneke, Cauê Vazquez La Scala Teixeira

**Affiliations:** ^1^Functional Training Group, Federal University of Sergipe, Aracajú, Brazil; ^2^School of Human Kinetics and Recreation, Memorial University of Newfoundland, St. John's, NL, Canada; ^3^Department of Health, Exercise Science, and Recreation Management, University of Mississippi, Oxford, MS, United States; ^4^Obesity Study Group (GEO), Federal University of São Paulo, Santos, Brazil

**Keywords:** blood flow restriction training, functional training, resistance trainig, hypertrophy, strength, conditioning

## Introduction

Alternative training methods to traditional high load resistance exercise have started to become more popular in the literature, such as functional (La Scala Teixeira et al., 2017) and blood flow restriction (BFR) training (Patterson et al., 2019). The main justification for the research involving these topics is to seek deeper understanding about the effects of techniques that may be used as substitutes for traditional high load resistance training. Although traditional high load training is effective for improving muscular strength and hypertrophy (Kraemer et al., 2002), it may not be the most suitable for all training environments such as musculoskeletal-, or cardiac-rehabilitation, calorie restrictions, among others.

In this context, low-load resistance training associated with BFR has been an efficient option when the goal is related to increased muscle strength and hypertrophy in situations that limit the use of high load training. Recent meta-analyses have revealed that the chronic effects on muscle mass of this type of training are similar to those in traditional high-load strength training, both in adults (Lixandrão et al., 2018) and in the older adults (Centner et al., 2019a). Both studies showed significant increases in strength, but BFR exhibited slightly lower training responses compared to the traditional model. These meta-analyses confirm that the method may be a viable alternative to heavy load resistance training. These same changes are also observed within trained individuals (i.e., athletes) (Takarada et al., 2002; Luebbers et al., 2014).

Functional training involves resistance training and associated techniques to develop strength, as well as balance, motor coordination, power and muscle endurance, increasing the ability of individuals to execute activities of daily living (ADL), whether they be simpler tasks of daily living or more complex athletic maneuvers. Functional resistance training has been promoted as a more training-specific option than traditional high-load strength training when the goal is to promote multi-systemic or multi-component adaptations, that is, to promote concomitant adaptations in different physical capacities (La Scala Teixeira et al., 2016, 2017). These results have been demonstrated in studies involving young adults (Distefano et al., 2013) and older adults (Liu et al., 2014). The rationale underlying multi-system adaptations with functional resistance training, can be partially explained by the manipulation of exercise complexity (i.e., progressive variations in stability, movement velocities, action, or task specificity) as the primary variable of training progression. More complex functional resistance exercises increase the demand upon physical abilities such as coordination, balance, agility, power, among others (La Scala Teixeira et al., 2019).

Considering that training with low-loads in combination with BFR contributes significantly to the increase of strength and muscle mass, and that functional training can provide improvement in skills such as balance, coordination, agility, power, endurance, and flexibility, the use of blood flow restriction associated with functional training appears as an interesting perspective for situations that require these adaptations concomitantly. However, this is a hypothesis that needs to be explored and confirmed in future studies.

Therefore, this opinion article provides a perspective view on the possible association of functional resistance training with blood flow restriction, presenting the main characteristics of both methods to provide a theoretical reference for the elaboration of future research projects.

## Blood Flow Restriction (BFR)

Low-load resistance exercise in combination with BFR has previously been shown to increase both muscle size and strength; with the gains in muscle size being similar to that of traditional high load resistance exercise (Fahs et al., 2015; Lixandrão et al., 2018). BFR training programs can be implemented with higher frequency programs (every day or even twice a day), resulting in strength and hypertrophy gains over a short intervention period (Abe et al., 2005; Fujita et al., 2008; Nielsen et al., 2012). Two purported ideas for the effects of blood flow restriction include cell swelling (Loenneke et al., 2012a) and heightened muscle activation due to the pooling of metabolites within the exercising muscle (Dankel et al., 2017). Both are thought to induce changes in gene expression and anabolic signaling similar to that of traditional high load resistance exercise (Gundermann et al., 2014; Ellefsen et al., 2015). Hence, BFR resistance training can be especially effective when high-loads are not possible or desirable in a training program.

Besides improving muscle strength and hypertrophy, BFR-induced physical capacity improvements have been demonstrated with several populations. Older adults may benefit from improved strength levels, muscle mass, and improved bone health (Cardoso et al., 2018). Of note, although changes in bone mass are traditionally thought to occur from higher impact exercise, there is theoretical rationale for improvements with load exercise in combination with BFR (Loenneke et al., 2012b). Whether bone is favorably influenced, however, requires longer training interventions than the 6–12 weeks studies common to the BFR literature. Hypertensive and normotensive individuals may also benefit from BFR-associated training from the greater hypotensive effect than traditional training, especially 30–60 min post-training session (Domingos and Polito, 2018; Bonorino et al., 2019).

Any benefit of low load resistance training in combination with BFR should be discussed within the context of safety. Although there is always inherent risk, the important question is whether the application of BFR augments that risk over the same exercise without BFR. Two common concerns include the risk of muscle damage and blood clotting, neither of which appears to be augmented by the application of BFR (Clark et al., 2011; Loenneke et al., 2014). In addition, a recent review concluded that when BFR is applied appropriately, that there does not appear to be an increased risk of endothelial damage (Da Cunha Nascimento et al., 2019).

## Functional Training

Functional resistance training is also a contemporary training concept aimed at enhancing task or activity functionality; that is, the ability of human beings to perform the full set of everyday tasks with autonomy and safety. Considering that daily activities involve the submaximal and simultaneous demand of different physical abilities, the objective of functional training is to promote the synergistic, integrated, and balanced improvement of these abilities to maximize transferability. In other words, the objective of functional training is to promote multi-system or multi-component adaptations (La Scala Teixeira et al., 2017).

As the primary means for applying this concept, most research suggested the use of strength training and associated techniques (for example calisthenics, Pilates), not only with the traditional focus on strength and hypertrophy, but stimulating improvement of strength together with other physical abilities such as coordination, balance, power, agility, among others (Da Silva-Grigoletto et al., 2014; La Scala Teixeira et al., 2017).

One of the main characteristics of functional resistance training is the complexity strategy progression, that is, the change of the motor pattern to increase the level of the technical difficulty of the exercise. Characteristics such as multi-segmental, multi-planar, fast, integrated, acyclic, and unsteady movement execution are commonly explored in functional training programs (La Scala Teixeira et al., 2019). Previous studies demonstrated the potential of functional training for promoting multi-component adaptations. Distefano et al. (2013) compared the effects of an 8-week functional strength training program (called “integrated training” by the authors), with a traditional strength training program (called “isolated training” by the authors), in young adults. The authors reported that functional training enabled more comprehensive adaptations, integrating a greater variety of physical abilities compared to the traditional model. Furthermore, in older adults, Brandão et al. (2017) and Chaves et al. (2017) observed greater multi-component adaptations with functional training compared to traditional training. Therefore, functional training seems to be a viable alternative to the traditional strength training model when the final goal is the concomitant improvement of different physical abilities.

## Functional Training With Blood Flow Restriction: A Perspective View

In a recent systematic review, Clarkson et al. (2018) highlighted an important research gap with BFR. The authors concluded that BFR-associated training has mostly focused on muscle size and strength with considerably less focus being given to possible benefits with activities of daily living and associated physical functions such as dynamic balance and mobility. Considering that BFR has a number of benefits for clinically limited populations (i.e., muscle hypertrophy with lower resistance loads, less muscle damage), they suggested BFR in combination with resistance training can provide an additional training stimulus to improve physical function that would be equivalent or superior to traditional resistance exercise. The integration of functional resistance training (multi-system, training-specific strength, and power adaptations) with BFR (increases in maximal strength and muscle mass) may be interesting to maximize the effects of isolated training.

Regarding the external overload used in functional training, the adoption of a percentage of 1RM (as observed in studies on traditional strength training) is not a common practice both in scientific studies or professional practice. Thus, our suggestion for functional training with BFR is to reduce ~70% of the load used in exercises under normal conditions (without BFR). This suggestion is based on the percentage of load reduction commonly used in studies that compare traditional strength training with high loads and strength training with low loads associated with BFR (Laurentino et al., 2012; Centner et al., 2019b).

In respect of blood flow restriction, as observed in most of the method-related publications for BFR, the use of pneumatic tourniquets with pressure gauges has been suggested to promote partial vascular occlusion as it provides objective pressure control and it is a key feature to ensure efficacy and safety in interventions (Mattocks et al., 2018; Patterson et al., 2019). However, pneumatic tourniquets are more expensive, have limited practicality and restrict the ability to perform more complex movements (Wilson et al., 2013).

In acyclic, asymmetric, multi-segmental, and multi-planar tasks (movements or exercises) such as those applied in the functional training, freedom of movement, and the inclusion of implements that allow such freedom in the training session is essential. Elastic tourniquets are a convenient, low cost, viable option allowing freedom of movement (Wilson et al., 2013; Lowery et al., 2014; Luebbers et al., 2014; Bell et al., 2018), enabling the integration of functional training with BFR. Of note, the precise monitoring of cuff pressures may not be necessary for muscular adaptations assuming the load used for exercise is not too low (Lixandrão et al., 2015). This is because comparable muscle adaptations are often observed across a wide range (40–90% arterial occlusion pressure) of applied relative pressures (Lixandrão et al., 2015; Counts et al., 2016; Jessee et al., 2018). In other words, similar changes in functional performance could be expected from lower load exercise completed with an elastic tourniquet applied with a moderate pressure or applied with a high pressure.

Evidence guiding the use of elastic tourniquets in strength training programs (Yamanaka et al., 2012; Wilson et al., 2013; Lowery et al., 2014; Luebbers et al., 2014), show that the properly controlled use of this implement can promote strength and muscle mass increases, and can be safe in young adults. One limitation with the use of practical blood flow restriction is the inability to know the applied pressure. One method proposed was to use a rating of “7” out of 10 on a perceived tightness scale (Wilson et al., 2013). This method is able to provide a rating below the arterial occlusion pressure (Bell et al., 2018), however, this scale is not reliable across time. In other words, the pressure associated with “7” out of 10 is not consistent from day to day limiting its use in training studies (Bell et al., 2019). On the other hand, a recent paper has suggested that applying a cuff/wrap at a percentage of the arm circumference may provide estimates similar to that of traditional methods of BFR (Abe et al., 2018). Another alternative procedure described by Behringer et al. (2017) was to pull the elastic wraps maximally around the limb and then mark at each quarter of every winding. Thereafter, the wraps were re-applied at 75% of their maximum tension. The best method for applying practical BFR requires further study.

Taken together, if proper care is taken, the use of an elastic tourniquet to promote BFR in functional training may become a viable option for maximizing adaptations related to strength and hypertrophy in this training type. Considering that isolated functional training is already effective in promoting multi-system adaptations, its association with BFR may have the potential to maximize such adaptations (Table 1). These hypotheses need to be tested in future investigations.

**Table 1:** Main possible adaptations from BFR low load resistance training, functional training and associations of techniques.

**Variables**	**Main possible adaptations**		
	**BFR low load resistance training**	**Functional resistance training**	**BFR + functional resistance training (?)**
Maximum strength	↑↑	↑↑	↑↑↑
Hypertrophy	↑↑↑	↑	↑↑↑
Muscle endurance	↑↑↑	↑↑	↑↑↑
Power	↑	↑↑↑	↑↑↑
Balance	-	↑↑↑	↑↑↑
Coordination	-	↑↑↑	↑↑↑
Agility	-	↑↑↑	↑↑↑
Flexibility	-	↑↑↑	↑↑↑
Velocity	↑	↑↑↑	↑↑↑
Cardiorespiratory fitness	↑	↑↑	↑↑

## Final Considerations

In the literature, BFR is associated with a variety of resistance training methods. Therefore, it is available as a tool to increase the internal training load for a spectrum of training modalities. The present perspective expounds upon the advantage of integrating BFR's reported enhancement of strength, hypertrophy, and power with lower resistive loads with the training specific advantages of functional resistance training. Hence, based on the prior literature, it is quite reasonable to suggest an integration of BFR with functional resistance training to enhance resistance training adaptations.

This opinion article aims to present the scientific community and fitness enthusiasts with a perspective on a possible future intervention based on the integration of two promising training techniques. The evidence presented in this article aims to generate scientific debate, about the current panorama of technical alternatives to traditional training methods.

## Author Contributions

All authors listed have made a substantial, direct and intellectual contribution to the work, and approved it for publication.

## Conflict of Interest

The authors declare that the research was conducted in the absence of any commercial or financial relationships that could be construed as a potential conflict of interest. The reviewer HC declared a past co-authorship with one of the authors DB to the handling editor.
